# Links of Short Physical Performance Battery Score with Incident Dementia: Results from the NHATS

**DOI:** 10.1093/geroni/igab046.1687

**Published:** 2021-12-17

**Authors:** Di Wu, Emily Smail, Jennifer Schrack, Amal Wanigatunga, Judith Kasper, Adam Spira

**Affiliations:** 1 Department of Mental Health, Johns Hopkins Bloomberg School of Public Health, Baltimore, Maryland, United States; 2 Department of Epidemiology, Johns Hopkins Bloomberg School of Public Health, Baltimore, Maryland, United States; 3 Johns Hopkins Bloomberg School of Public Health, Baltimore, Maryland, United States; 4 Department of Health Policy and Management, Johns Hopkins Bloomberg School of Public Health, Baltimore, Maryland, United States

## Abstract

Physical performance is associated with cognitive function in later life, but few studies have examined the prospective association of physical performance with incident dementia. We studied 4539 community-dwelling National Health and Aging Trends Study (NHATS) participants aged ≥65 years with data on demographics and the Short Physical Performance Battery (SPPB) in 2011, who were followed through 2014. Our outcome was dementia diagnosis from a validated NHATS algorithm. We applied survey weights to make results nationally representative and performed Cox regression analyses. After adjustment for potential confounders, lower baseline SPPB scores were associated with incident dementia (HR=1.68, p < 0.01). Slower gait speed was the SPPB component most strongly associated with incident dementia (HR=1.21, p < 0.01). We found that poorer physical performance was linked to incident dementia in a cohort of older adults. More research is needed to examine the effect of improving physical performance on the prevention of dementia.

